# Stress Exacerbates Infectivity and Pathogenicity of *Blastocystis hominis*: In Vitro and In Vivo Evidences

**DOI:** 10.1371/journal.pone.0094567

**Published:** 2014-05-02

**Authors:** Samudi Chandramathi, Kumar Suresh, Sinnadurai Sivanandam, Umah Rani Kuppusamy

**Affiliations:** 1 Department of Parasitology, Faculty of Medicine, University of Malaya, Kuala Lumpur, Malaysia; 2 Department of Biomedical Science, Faculty of Medicine, University of Malaya, Kuala Lumpur, Malaysia; University at Buffalo, United States of America

## Abstract

**Background:**

Stress alters the oxidant-antioxidant state and immune cell responses which disrupts its function to combat infection. *Blastocystis hominis*, a common intestinal protozoan has been reported to be opportunistic in immunocompromised patients namely cancer. *B. hominis* infectivity in other altered immune system conditions especially stress is unknown. We aimed to demonstrate the stress effects towards the susceptibility and pathogenicity of *B. hominis* infection.

**Methods/Findings:**

Three-week-old Wistar rats were divided into four groups: (a)control; (b)stress-induced; (c)*B. hominis* infected; (d)stress-induced with *B. hominis* infection; (n = 20 respectively). Stress was induced for an hour daily (30 days) using a Belly Dancer Shaker. Weight gain was monitored, stool samples were collected for *B. hominis* screening and blood for the determination of differential count, levels of immunoglobulin, oxidative damage, and peripheral blood mononuclear cell (PBMC) proliferation upon induction with solubilized antigen of *B. hominis* (Blasto-Ag). Group (b) exhibited the highest level of weight gain. Group (d) had higher levels of parasite cyst count in stools, serum IgE, oxidized protein and lipid compared to the group (c). Levels of monocyte and antioxidant in group (d) were decreased and their PBMCs showed highest inhibition of proliferation level when exposed to Blasto-Ag. Monocyte level in Group (b) showed insignificant difference compared to group (a) but was significantly lower compared to group (c). Antioxidant levels in group (c) were generally lower compared to group (a) and (b). Inhibition level exhibited by Blasto-Ag treated PBMCs of group (c) was higher compared to group (a) and (b).

**Conclusion:**

The pathogenicity and augmentation of *B. hominis* infection is enhanced when stress is present. Lifestyles today are becoming increasingly stressed and the present findings suggest that the parasite which has been reported to be one of the most common organisms seen in stool surveys, namely in developing countries, may tend to be more pathogenic in stressful situations.

## Introduction

In today's world, humans are often impacted with stress in the course of pursuing success in carrier, wealth and survival. In United States of America, reports have shown that in the year 2009 stress increased 24% and 18% for both man and women respectively as compared to the year 1983 [1]. Stress is defined as a type of condition or response in a living being that is caused by various types of internal or external stimulus [2]. Stress is known to cause behavioral and psychological changes that can lead to disturbance in the body's physiological function including imbalance in the oxidant-antioxidant state [3]. The overproduction of oxidants or free radicals such as superoxides (O_2_^•^) and hydroxyls (OH^•^) compared to the antioxidants will result in a state called oxidative stress. Oxidative stress is known to cause oxidation of lipid, protein and DNA of cells [4] which results in the abnormal function of tissues and organs of the body. A considerable number of studies done on humans and animals have gathered evidences to associate stress with the etiology of various psychotic and metabolic diseases such as depression, gastritis, rheumatoid arthritis, and cancer [5]–[7].

Previous studies done on rodents have also demonstrated that chronic and acute stress using vibration and restrain stressors can impair immune responses by altering the activities of peripheral blood mononuclear cells (PBMCs) such as lymphocytes, neutrophils and monocytes which will culminate in abnormal antibody productions [7], [8]. Disruption in the PBMCs function may affect its role in combating invading antigens or infections including intestinal parasitic infections. Generally, when a host's immune system is triggered by parasitic infection, a massive production of oxidants are activated by immune cells including PBMCs to eradicate the infection [9]. In long term infections, the continuous release of reactive species together with a lack of antioxidant production will result in oxidative damage, exposing the host to other illnesses.

Intestinal parasites such as *Ascaris lumbricoides* and *Cryptosporidium parvum* have been reported to cause alterations in the molecular functions of the hosts' PBMCs and immunoglobulin levels [10], [11]. In addition, antigens originating from intestinal parasites namely *Ascaris lumbricoides* and *Trichuris trichiura* have been shown to alter *in vitro* proliferation of PBMCs isolated from normal and parasite infected individuals [12]. The presence of oxidative stress and compromised antioxidant defence mechanisms in humans and experimental models infected with intestinal protozoans such as *Entamoeba histolytica* and *Giardia* sp. have also been reported previously [13]. However, to the best of our knowledge, there have been no reports on the role of stress in contributing to host's immunosuppression, oxidative damage, and susceptibility to intestinal parasitic infections.

*Blastocystis hominis*, is one of the most common intestinal protozoan parasites found in humans. It is known to show diverse morphologies and reproductive processes [14]. The prevalence of *B. hominis* ranges from approximately 10% in developed countries and up to 60% in developing countries [15]. The extreme dispute regarding its pathogenicity had led to the remarkable findings on both phenotypic and genotypic characteristics of asymptomatic and symptomatic human-derived *B. hominis* isolates [16]. Various reports have shown that certain subtype of this unicellular protozoan is coupled with intestinal disorders including Irritable Bowel Syndrome (IBS) [17]. Previously, we have reported on the elevation of oxidative damage and proinflammatory cytokines caused by *B. hominis* infection in animal model [18], [19]. We have also demonstrated that solubilized antigen from *B. hominis* (Blasto-Ag), at a certain concentration, could down-regulate PBMC responses while enhancing the growth of colorectal cancer cells *in vitro* [20]. Additional comparative study revealed that Blasto-Ag derived from symptomatic individual has higher inhibitory effect on PBMCs, implying a higher pathogenicity (our unpublished data). Our recent study showed that infection of *B. hominis* in an immunocompromised condition such as cancer is opportunistic [21]. However, the infectivity of this parasite in other immune-system-altered condition such as in stress is not known.

Therefore, the main aim of this study is to provide experimental evidence on how chronic stress influences the hosts' immune system, causes oxidative damage, and increases susceptibility towards *B. hominis* infection. To achive this, we used mechanical stress (by shaking) which is a simple and convenient model to induce both psychological (shocked reaction) and physical (body muscle movements) stress in rats prior to exposing them to symptomatic *B. hominis* infection. The effects of Blasto-Ag (isolated from symptomatic human) on the *in vitro* growth of PBMCs isolated from rats subjected to stress and *B. hominis* infection were also investigated. This experimental model is hoped to lead to a better understanding of the immune cell responses towards *B. hominis* infection in humans with and without stress.

## Materials and Methods

### Source and Isolation of *B. hominis* cysts

*B. hominis* was isolated from the stool samples of a symptomatic human and the cysts were extracted using Ficoll-Paque technique with a slight modification [22]. Isolated cysts were washed in sterile saline and incubated at room temperature for two days in saline added with 100units/ml penicillin-streptomycin. These steps were repeated two to three times prior to the inoculation stage, in order to eliminate the possible bacterial contamination. Furthermore, the cysts were further cultured in Jones' medium to examine the growth of parasite. Only purified cysts without any bacterial contamination were used for the inoculation.

### Ethics statement

Prior to stool sample collection, a written informed consent was obtained from all recruited individuals. The protocol used in this study was approved by the Medical Ethics committee of the UMMC (University Malaya Medical Center) which complies with the Declaration of Helsinki. The animal care and research protocol used in this study was approved by University of Malaya Institutional Animal Care and Use Committee (UM IACUC) according to The National Research Council's Guide for the Care and Use of Laboratory Animals (8th edition). Our ethics reference number is PAR/30/03/2012/SKG(R).

### Inoculation of *B. hominis* cysts and stress induction in animal model

Three-week-old Wistar albino rats with a mean weight of 60 grams were assigned into four groups comprising 20 rats in each group namely a) control (without stress induction and without *B. hominis* infection), b) stress-induced, c) *B. hominis* infected and d) stress-induced and *B. hominis* infected. Weights of all rats were recorded once a week to examine changes in their weight gain. The 20 rats in each group were housed into 4 cages of 5 rats. Throughout the experiment, it was made sure that the rats from each study group were kept separated in appropriately labelled cages. A week prior to the commencement of the experiment, the rats were pre-screened in order to ensure that they were healthy and free of *B. hominis* or any other intestinal parasitic infection. Ten thousand cysts in 1 ml sterile saline (without penicillin-streptomycin) were orally inoculated to each rat in the infected group. The control group was inoculated with 1 ml of sterile saline. For the stress-induced group, the respective cages of rats were placed on a Belly Dancer Shaker which was shaken at a speed of 90 rpm for 1 hour on a daily basis for a period of 30 days. The undulating motion of the Belly Dancer Shaker provided gentle agitation and induced stress to the animals especially when the speed was increased gradually (done within 5 minutes prior to shaking period). The combination of both vertical and horizontal orbital movements provided by the Belly Dancer Shaker would be able to mimic physical stress better than other types of laboratory shakers namely orbital shaker.

### Detection of *B. hominis* in stool samples

Stool samples of all rats were examined for the presence of *B. hominis* daily till the 14th day and then after on alternate days till the last day of experiment. Briefly, three pellets of fresh stools were emulsified in normal saline (3–5 ml) and filtered using gauze to eliminate debris. The filtrate containing cysts were then washed 3 times with PBS. After the final washing step, supernatant was discarded and the pellet was re-suspended with minimum volume of PBS (10 µl to 50 µl). A drop of this mixture (10 µl) was mixed with 10 µl of trypan blue stain and cyst count was performed using haemocytometer and light microscope. Based on the concentration of cysts determined in this step, the number of *B. hominis* cysts in the PBS mixture (10 µl to 50 µl) was recalculated and expressed as cyst/ml (the final volume of the PBS mixture was adjusted to 1 ml). Concurrently, stool samples were also cultured in Jones' medium and observed after 24 hours to confirm the viability of this parasite [23].

### Blood sample collection

Blood samples (from five rats per group) were collected once a week using heart-puncture technique. Prior to blood sampling, each rat was placed in a covered cylindrical jar and anesthetised with diethyl ether until loss of the righting reflex which takes approximately 75 seconds. Any possible anesthetic influence on the results was reduced by using a standardized euthanizing technique to all the animals including the control group. After collecting the blood samples, rats were euthanized by overdosing with diethyl ether. Blood samples were collected in plain and EDTA coated tubes to obtain serum and whole blood respectively. Serum sample was used for biochemical and immunoglobulin analysis whereas whole blood was used for PBMC isolation and blood differential count.

### Biochemical assays

Advanced oxidation protein product (AOPP) is a biomarker used for free radical induced protein damage. It is formed by the action of chlorinated oxidants especially hypochlorous acid and chloramines. The level of AOPP was measured spectrophotometrically according to an established method [24]. The level of AOPP was calculated based on a standard curve generated using chloramine T and the results were expressed as µM chloramine T equivalents.

Lipid hydroperoxide (LHP) is the intermediary product in free radical induced lipid peroxidation. LHP level was assessed according to the method by Esterbauer and Cheeseman [25], with minor modification. In this method, the LHP present in a biological sample reacts with 1-methyl-2-phenylindole (MPI) under acidic condition to form a blue-coloured chromophore which can be measured spectrophotometrically. The level of LHP was estimated using 1,1,3,3- tetraethoxypropane as a standard and the results were expressed as µM.

The non-enzymatic antioxidants or reductants in serum samples were assessed using the ferric-reducing antioxidant power (FRAP) assay, a method previously established by Benzie and Strain [26] and further modified in our laboratory. Antioxidants present in sample reduce ferric ion-tripyridyltriazine (Fe2+-TPTZ) to ferrous ion-tripyridyltriazine (Fe3+-TPTZ) at low pH causing the formation of blue-coloured ferrous-tripyridyltriazine complex which can be measured spectrophotometrically. Concentration of reductants was determined using ferrous sulphate heptahydrate (FeSO4 7H2O) as the standard and results were expressed as µM.

Glutathione peroxidase (GPx), an enzymatic antioxidant was assayed using quantitative colorimetric assay kit purchased from BioAssay Systems, USA. Assay buffer, glutathione, NADPH, and glutathione reductase enzyme were mixed together with serum sample in a 96-well plate. Finally, hydrogen peroxide (H2O2) which is the substrate for GPx enzyme was added and the absorbance at 340 nm was taken at 0 and 4th minute. This assay directly measures the NADPH consumption in the enzyme coupled reactions. The decrease in optical density is directly proportional to the enzyme activity in the sample. GPx activity (U/L) was calculated using a NADPH standard curve as recommended by the assay kit's user manual.

### Blood Differential Count

A thin blood film (from ethylenediaminetetraacetic acid or EDTA tube) was prepared on a microscopic glass slide. In order to easily visualize the differences among the various types of WBCs, the blood film was stained with Giemsa stain [27]. Briefly, one hundred WBCs were counted and the percentage of the various WBC components namely neutrophils, eosinophils, lymphocytes, and monocytes were calculated.

### Isolation of Peripheral Blood Mononuclear Cells (PBMCs)

Whole blood sample (approximately 2 ml) was collected in sterile EDTA-tubes. PBMCs were harvested using Histopaque-1077 (Sigma-Aldrich, USA) according to the gradient density centrifugation technique [28]. The isolated PBMCs were washed thrice with phosphate buffer saline (PBS) and transferred into growth media (Roswell Park Memorial Institute medium or RPMI-1640 supplemented with 10% Fetal Bovine Serum or FBS, 2 mM L-glutamine, 100 units/ml penicillin-streptomycin, and 2·5 µg/ml fungizone).

### Isolation of *B. hominis* solubilized antigen (Blasto-Ag) and introduction to PBMCs

Axenization of *B. hominis* and isolation of Blasto-Ag were done according to the method used in our previous study [20]. Briefly, the axenized parasites were transferred into sterile Jones' medium (without any supplements) and lysed using sonication technique before incubating overnight at 4°C. The homogenate was filter sterilized and the protein concentration was determined by Bradford assay. Freshly isolated PBMCs (5×10^4^ cells/well) in 100 µl growth medium were seeded into 96 well plates. After an overnight incubation at 37°C in a CO_2_ incubator containing 5% CO_2_, Blasto-Ag with a final concentration of 1 µg/ml was added into each well and was further incubated for 48 hours. The concentration of Blasto-Ag used in this study was based on our previous published data [20] which caused optimum proliferation of PBMCs isolated from healthy individual. In addition to this, preliminary experiment using PBMCs of 5 normal rats exposed to various concentration of Blasto-Ag (0.001 to 10 µg/ml) showed that 1 ug/ml resulted in optimum cell proliferation (our unpublished data). The changes in proliferation percentage of cells were measured using MTT (3-[4,5-dimethylthiazol-2-yl]-2,5 diphenyl tetrazolium bromide) assay as described previously [29]. This colorimetric assay is based on the conversion of yellow MTT dye into purple formazan crystals by the mitochondrial enzyme activity of living cells. The formazan crystals were dissolved using DMSO and the absorbance was measured at 700 nm against 560 nm.

### Measurement of Immunoglobulins IgG, IgE, and IgM in serum samples

Immunoglobulins namely IgG, IgE, and IgM were measured according to the user manual of Enzyme–Linked Immunosorbent Assay (ELISA) kit purchased from Immunology Consultants Laboratory, USA. Briefly, IgG, IgE or IgM present in serum samples combined with the respective anti-immunoglobulin antibodies that have been coated on the surface of polystyrene microtitre wells (96-well plate). The unbound proteins were removed by a washing step and the addition of anti-immunoglobulin antibodies conjugated with horseradish peroxidase (HRP), form complexes with the previously bound immunoglobulin. After another washing step, the chromogenic substrate, tetramethylbenzidine (TMB) was added resulting in the formation of coloured solution which was proportional to the quantity of respective immunoglobulin present in the serum samples. This reaction was inhibited using the provided ‘Stop Solution’ that caused a colour change from blue to yellow and the absorbance was measured at 450 nm. The level of immunoglobulins in the test sample was quantified using standard curve created according to the assay kit's user manual.

### Statistical analysis

All data were analyzed using SPSS version 18. Values are expressed as mean ± SD. The significant difference between the control (normal) and other groups were analyzed using Student t-test. Comparison of parameters tested among the ‘infected’, ‘stressed’, and ‘stressed and infected’ groups were done using one-way ANOVA analysis. Correlations between the parameters for both control and parasite infected animals were identified by Pearson's correlation coefficient test and differences were considered significant when P<0·05.

## Results

**Figure 1:**
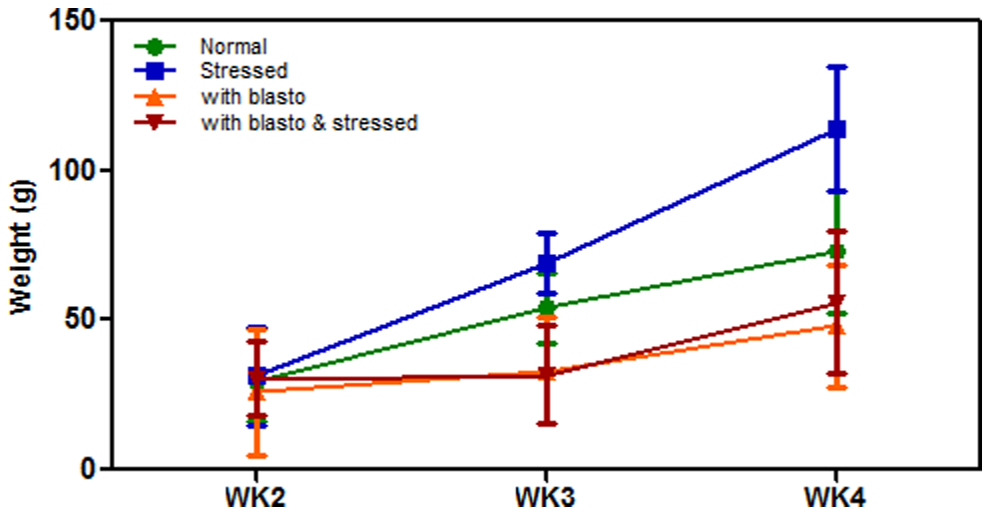
Effects of stress and *B. hominis* infection on weight gain. Mean weight gain is compared with their initial weight at week 1. Data shown is in mean ± SD. WK =  week.

**Figure 2:**
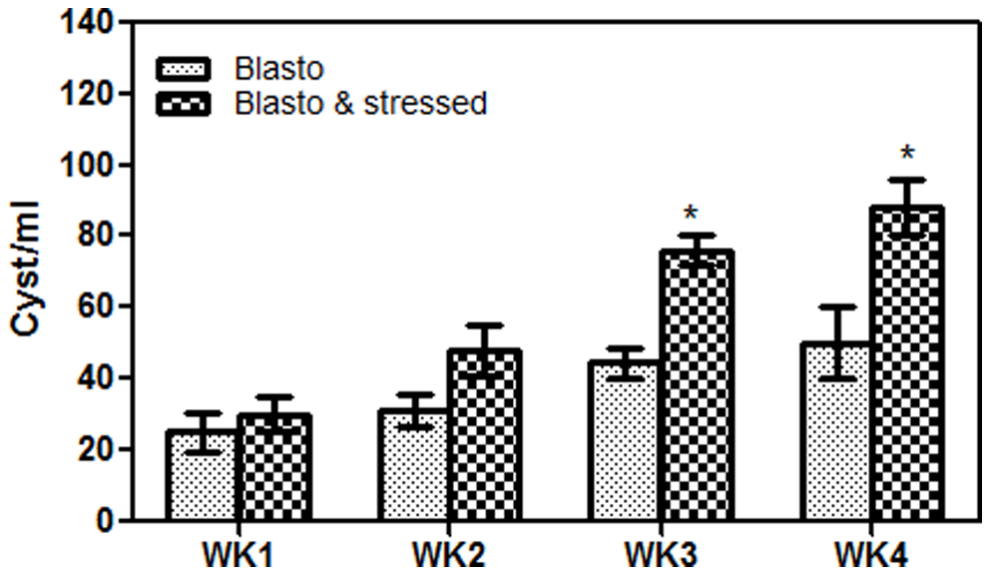
Cyst count in Blasto and Blasto-Stressed groups according to study duration. Data shown is total number of cysts recovered from 3 stool pellets from each rat (n = 5). Results are expressed as mean ± SD. *P<0.05 is the comparison done against Blasto group.

In present study, the control group (without stress and *B. hominis* infection) was labelled as ‘Normal’, stress-induced group as ‘Stressed’, *B. hominis* infected group (without stress) as ‘Blasto’, and both stress-induced along with *B. hominis* infection as ‘Blasto-Stressed’. The mean weight gained in each week for all groups were compared with week 1 (Figure 1). All groups showed a significant increase in weight gain at week 4 (P<0·05) when compared to week 2. Stressed group showed the highest level of weight gain followed by Normal, Blasto, and Blasto-Stressed. The cyst count in both Blasto and Blasto-Stressed groups showed a gradual increase over the four weeks (Figure 2). The Blasto-Stressed group had significantly higher number of cysts compared to the Blasto group at week 3 and week 4 (P<0·05 respectively). Irregular shedding of *B. hominis* has been reported previously [30]. It is noteworthy that we observed absence or irregular cyst shedding only in some of the rats especially during the first two weeks of the study. However, the irregular shedding effect among these two study groups were normalized by expressing the mean value of cyst count obtained from 5 rats per group/week.

**Figure 3:**
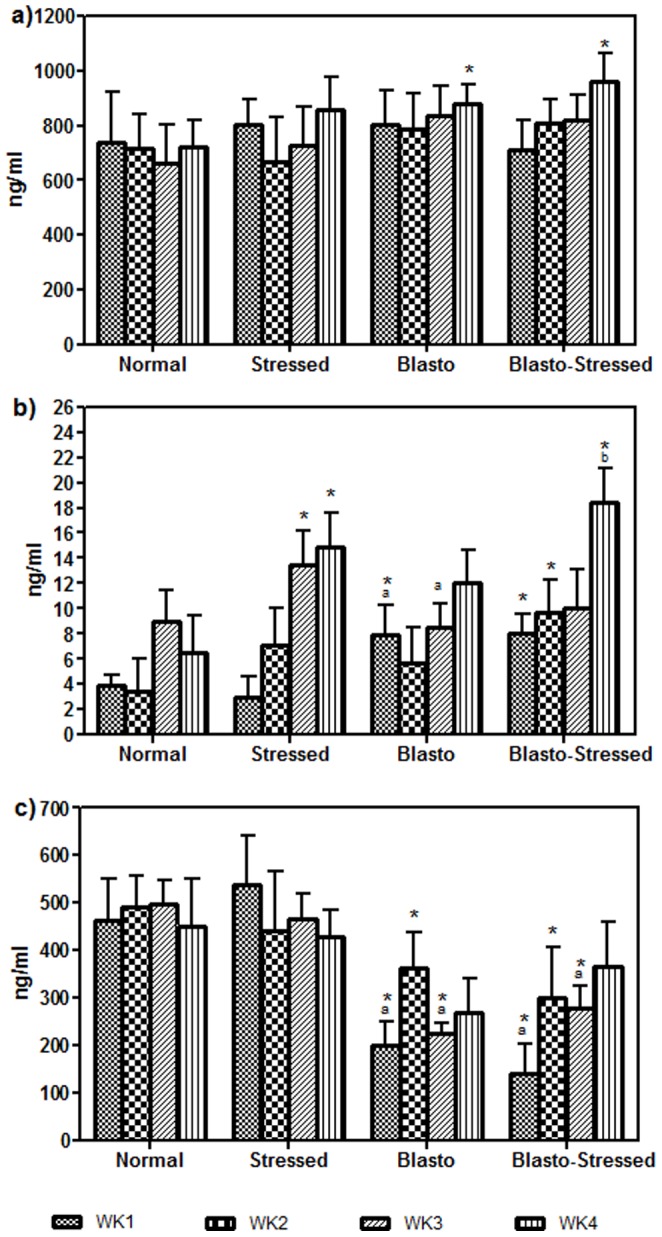
Levels of serum a) IgG, b) IgE, and c) IgM in the study groups according to study duration. Data is given in mean ± SD. *P<0.05 is the comparison done against Normal group using Student's t-test. ^a^P<0.05 is the significant comparison done against Stressed group using One-way ANOVA analysis.

The level of IgG did not significantly differ between Normal and Stressed groups when compared between week 1 and 4 (Figure 3a). However, an increasing trend was observed in the IgG level of Blasto and Blasto-Stressed groups with a significant difference when compared to the normal group especially at week 4 (P<0·05). The levels of IgE showed an increasing trend in all four groups (Figure 3b). In the final week of experiment, Blasto-Stressed group had higher level of IgE compared to Normal and Blasto groups (P<0·05 respectively). Besides this, the level of IgM in Blasto and Blsto-Stressed groups were generally lower when compared to Normal and Stressed groups with the significant difference seen mainly during weeks 1, 2, and 3 (P<0·05 respectively, Figure 3c).

**Figure 4:**
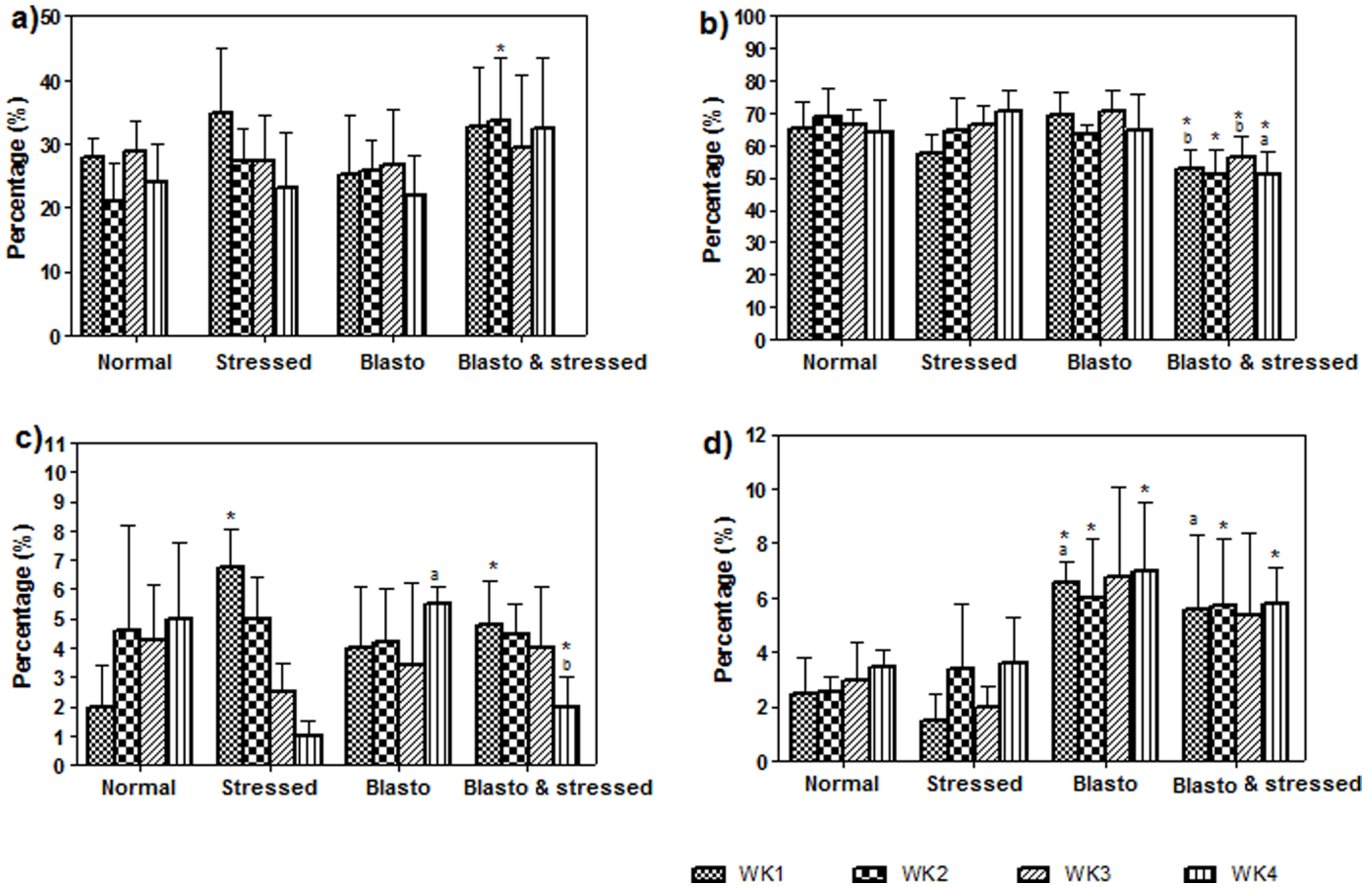
Blood differential count: a) neutrophil, b) lymphocyte, c) monocyte and d) eosinophil in the respective group of rats according to study duration. Data is given in mean ± SD. *P<0.05 is the comparison done against Normal group by Student's t-test. ^a^P<0.05 and ^b^P<0.05 is the comparison done against Stressed and Blasto group respectively using One-way ANOVA analysis.

Results of blood differential count showed that the level of neutrophils in Blasto-Stressed group was higher but not significant compared to the other three groups (Figure 4a). In contrast, the level of lymphocytes in this group was lower compared to the rest of the study groups in particular the normal group (P<0·05, Figure 4b). Level of monocytes in the Stressed group did not show any significant difference when compared to the normal group. However, there was a gradual decrease in the monocyte level of Stressed group from week 1 to week 4 (Figure 4c). During the final week, the level of monocytes in Blasto-Stressed group was significantly lower compared to Normal and Blasto groups (P<0·05). Besides this, the level of eosinophils in Blasto and Blasto-Sressed groups were significantly higher compared to normal group (P<0·05 respectively) especially in week 4 (Figure 4d).

**Figure 5:**
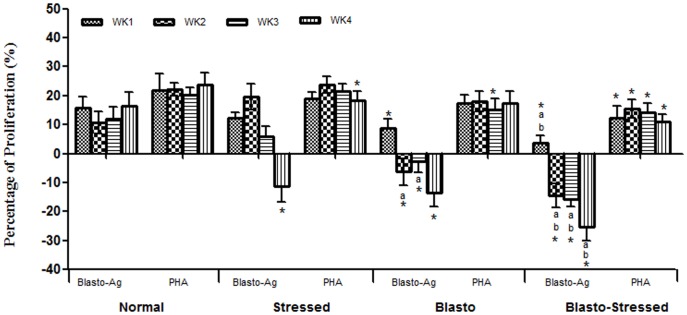
Proliferation of PBMCs introduced with 1 µg/ml of Blasto-Ag according to study duration. PHA, mitogen (20 µg/ml) was used as positive control. Values are given in mean ± SD. Values are normalized against sample blank where Blasto-Ag was substituted with sterile Jones medium (without any supplements). ^*^P<0.05 is the comparison done against Normal group by Student's t-test. ^a^P<0.05 and ^b^P<0.05 is the comparison done against Stressed and Blasto group respectively using One-way ANOVA analysis.

Figure 5 depicts the effect of Blasto-Ag on the proliferation of PBMCs isolated from rats belonging to the respective groups. PBMCs isolated from Blasto-Stressed group showed the highest inhibition (P<0·05) when exposed to Blasto-Ag and this was followed by the Blasto and Stressed groups.

**Figure 6:**
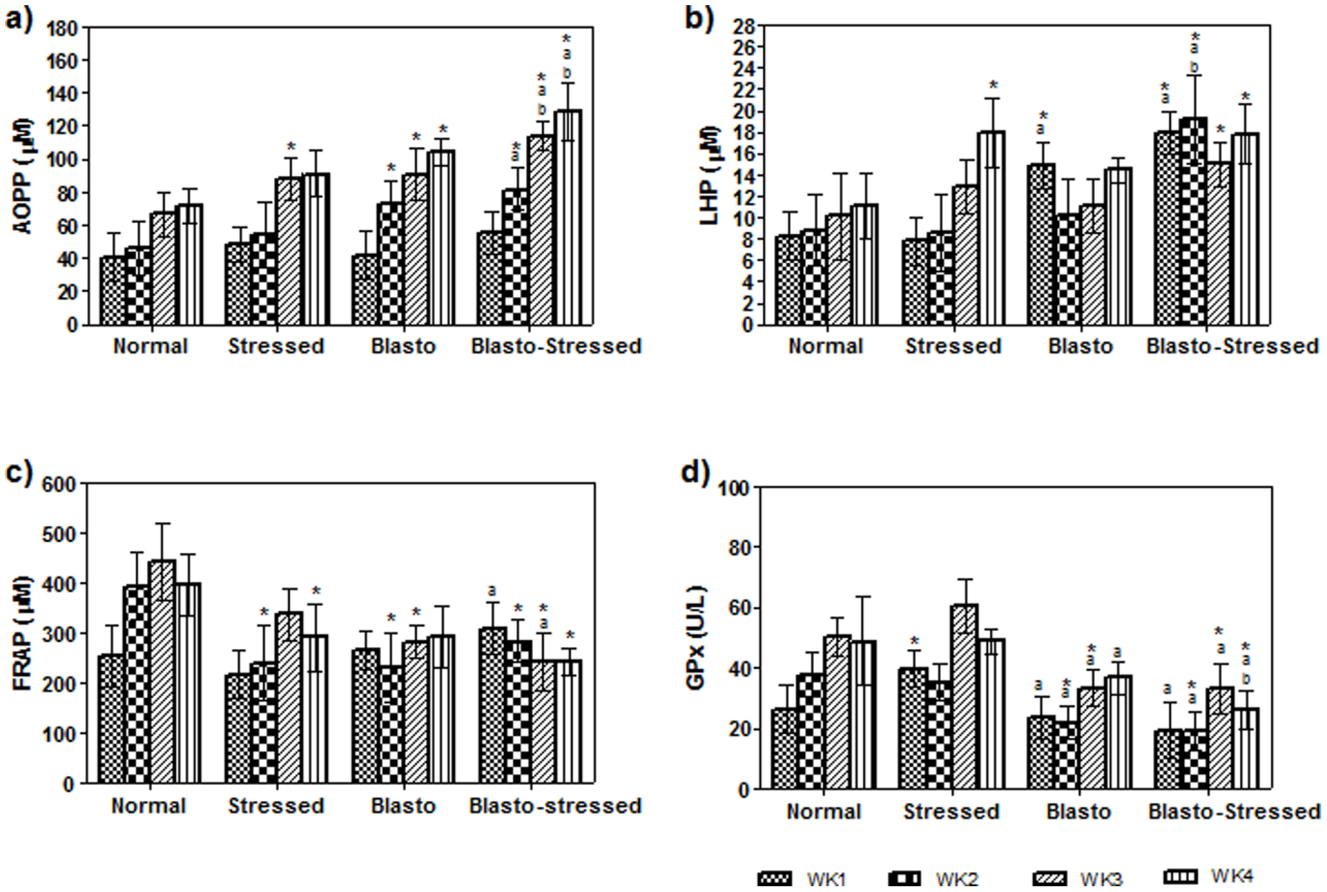
Levels of serum oxidative indices: a) AOPP, b) LHP, c) FRAP and d) GPx in the study groups according to study duration. Data is given in mean ± SD. *P<0.05 is the comparison done against Normal by Student's t-test. ^a^P<0.05 and ^b^P<0.05 is the comparison done against Stressed and Blasto group respectively using One-way ANOVA analysis.

Figure 6 depicts the levels of AOPP, LHP, FRAP, and GPx in all the groups according to the weeks. Level of AOPP was significantly elevated in Stressed, Blasto, and Blasto-Stressed groups compared to the normal group especially in week 3 and 4 (P<0·05 respectively). Blasto-Stressed group showed the highest level of AOPP especially in the last two weeks of the experiment (Figure 6a). Similarly, level of LHP was the highest in Blasto-Stressed group compared to other study groups and the significant difference (P<0·05) was evident mainly in week 1 and 2 (Figure 6b). In contrast, FRAP level in all the three experimental groups was lower compared to the normal group (Figure 6c). FRAP level of Blasto-Stressed group was significantly higher than Stressed group in week 1 but decreased significantly in week 3 (P<0·05 respectively). GPx level in Blasto and Blasto-Stressed groups were generally lower compared to normal and stressed groups. During the final week of experiment, rats in the Blasto-Stressed group exhibited the lowest level of GPx compared to all other groups (P<0·05).

Correlation analysis of AOPP, LHP, FRAP, and GPx levels in the study groups are shown in Table 1. In the Stressed group, most of the parameters showed positive correlation. In Normal group, only FRAP and GPx correlated positively whereas in Blasto group, positive correlation existed between AOPP and GPx. Negative correlation between AOPP and FRAP was evident in Blasto-Stressed group.

**Table 1:** Correlation analysis of AOPP, LHP, FRAP and GPx levels in the study groups.

Group	Parameters	Correlation
Normal	FRAP/GPx	r = 0·646, p<0·01
Stressed	AOPP/LHP	r = 0·660, p<0·01
	AOPP/FRAP	r = 0·521, p<0·05
	AOPP/GPx	r = 0·663, p<0·01
	FRAP/GPx	r = 0·590, p<0·01
Blasto	AOPP/GPx	r = 0·550, p<0·05
Blasto-Stressed	AOPP/FRAP	r = −0·591, p<0·01

Pearson's correlation coefficient test; differences were considered significant when p<0.05.

## Discussion

Infection of *B. hominis* in humans is known to cause clinical symptoms such as diarrhea, anorexia, flatulence, nausea, and abdominal discomfort [31]. Its pathogenicity which is a debatable topic has stimulated interest among researchers to study the epidemiological and molecular aspects of this protozoan. Various reports have shown the association between its pathogenicity, with genotypic characteristics and serious illnesses namely Irritable Bowel Syndrome as well as colorectal cancer. To date this is the first study to report on the effect of stress on the infectivity and pathogenicity of *B. hominis* infection in an animal model.

**Figure 7:**
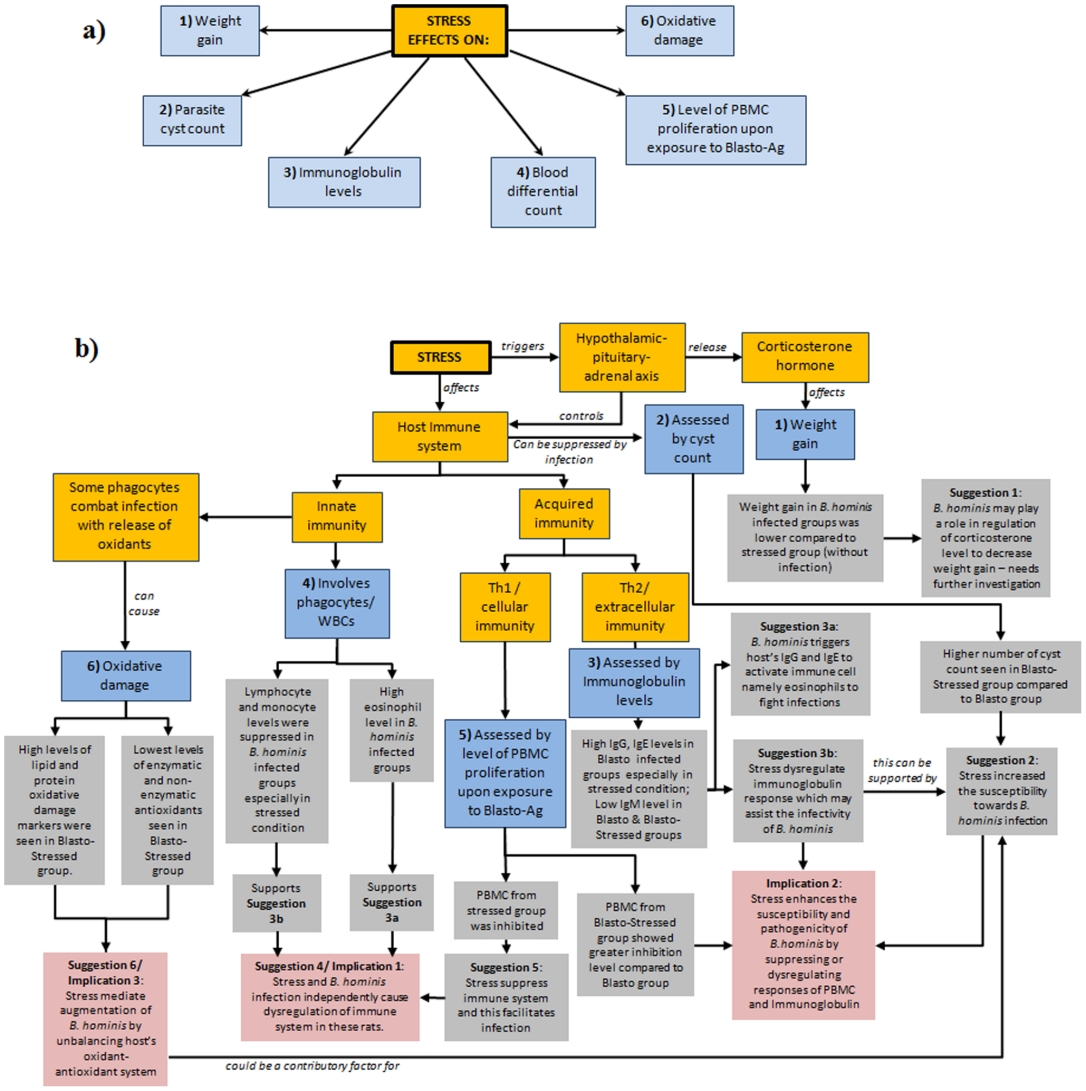
Illustration on the a) effects of stress on various parameters and b) overview of results obtained from this study. Text box shaded in a) yellow represents general facts and hypothesis, b) blue represents the parameters used, c) grey represents observations obtained, and d) pink represents implications of the current study.

In the present study, the physical stress (induced by Belly Dancer Shaker) introduced to the rats is considered a form of stress as these rats are unfamiliar to such agitation and is not their normal living condition. This could disrupt their homeostasis system which will eventually result in oxidative stress as well as immune system malfunction. Alteration in the immune cell responses will destroy the efficiency to fight against infection. Here, we studied the effects of stress against the infectivity and pathogenicity of the common intestinal parasite, *B. hominis*. The effects were evaluated using several parameters namely weight gain, cyst count, alteration in immunoglobulin levels, alteration in blood differential count, PBMC response *in vitro* and the level of oxidative indices as summarized in Figure 7a. Results obtained are discussed according to the number order (in the blue boxes) which indicates the experimental flow.

Stress response involves a range of biological and behavioral paths which is important for the survival or adaptations to changes in the environmental conditions. Stress triggers the hypothalamic-pituitary-adrenal axis to release glucocorticoid hormone (exist predominantly as corticosterone in rodents and cortisol in humans) that regulates the cascade of metabolic pathways in order to retain the internal physiological balance [32]. A study done on human subjects has reported that stress can cause both increase and decrease in weight or food intake [33] that has been linked with the level of glucocorticoid hormone. In current study, rats in Stressed group exhibited the highest level of weight gain compared to Normal and the other two groups (Figure 1). In contrast, researchers have also reported that restraint stress on rats resulted in decreased weight gain compared to the controls [34]. Another study has reported that only variable stressors (such as food deprivation, isolation, and swim stress) caused weight loss in rats compared to the chronic stress [35]. Similar to our findings, Badiani and colleagues [36] have reported on the significant increase in food intake in rats subjected to only a brief restraint period. However, the stress exposure period in all those studies were relatively short, ranging from 7 to 20 days and the effect of infection in the animals were not investigated. In our study, decrease in weight gain compared to the control rats were only observed when *B. hominis* infection was present (Blasto and Blasto-Stressed groups). Since the corticosterone level was not measured as a stress indicator we are unable to rule out the possible influence of *B. hominis* infection especially in the presence of stress on this glucocorticoid hormone. In view of the numerous previous studies which support the notion that the corticosterone level might not be a reliable marker of chronic stress, the exclusion of this hormone in the present study is well justified. Previous study has reported that increase in plasma corticosterone was independent of reduced food intake in rats experimentally infected with *Nippostrongylus brasiliensis*, a gastrointestinal nematode of rats [37]. Additionally, another study suggested that it is possible to claim that chronic stress may not directly affect the level of serum or plasma glucocorticoids [38]. The key point here is the response of target tissues or organs and the resulting oxidative damage rather than the levels of the glucocorticoid stress hormone. In view of the fact that previous evidences have shown insignificant change in the levels of glucocorticoid as well as the presence of glucocorticoid receptor resistance (GCR) in humans and(or) animals exposed to chronic stress [39], [40], it was more feasible to measure the end-products of oxidative damage as indicators of oxidative stress. Furthermore, researchers had studied the effect of chronic stress on oxidative damage parameters in rats without measuring corticosterone level [41]. Nevertheless, it may be feasible to include corticosterone level in brain or other targeted organs in future studies pertaining to chronic stress and its effect on HPA (Hypothalamic–pituitary–adrenal) axis.

Generally, a high cyst count is an indication of a very high number of trophozoites (which have reverted to become cysts) in the intestinal tract which are continuously multiplying through binary fusion. In addition to this, an increase in cyst count has been associated with the capability of a parasite to proliferate by escaping from host immune response. Studies have therefore correlated high number of cysts with susceptibility towards an infection [42]. A higher level of cyst count in the Blasto-Stressed group compared to the Blasto group observed in our study (Figure 2) has clearly suggested that stress increased the susceptibility towards *B. hominis* infection by suppressing the hosts' immune system. It is established that stress leads to the suppression of immune cell responses which inevitably creates a favorable condition for the growth of infectious microorganisms [43] including *B. hominis*. Generally, immune system in our body combats the invading antigens or parasites by two types of responses namely innate and acquired immunity [9]. The innate immunity involves natural killer (NK) cells and phagocytes or white blood cells (e.g.:monocytes, neutrophil, eosinophil) which eradicate the antigens by phagocytosis. The acquired immunity involves T helper (Th) lymphocytes that can be subdivided into Th1 and Th2 responses, also known as cellular and humoral immunity respectively. Th1 or cellular immunity is the defense against intracellular pathogens. In contrast, Th2 or humoral immunity plays the major role in defense against extracellular pathogens by triggering the production of immunoglobulin subtypes such as IgG, IgE, and IgM.

Generally, IgG makes up 80% of our circulating immunoglobulins and it is the most versatile immunoglobulin to act against invading pathogens. Its activity involves phagocytosis and activation of complement system to eradicate infection. IgE is secreted by plasma cells of mucous membrane and has been often associated with inflammatory responses. Increase in total serum IgE has been reported in humans with intestinal parasitic infection [44], [45]. Meanwhile IgM, an antibody found on the B cell surface is the first antibody produced to fight against an infection by involving humoral immunity [9].

Based on their immunological functions IgG, IgE and IgM have been previously assessed to understand the immune response of host infected with intestinal parasites [44], [46]. One of these studies [46] included the assessment of IgA which is known to be secreted by mucosal lining of gastrointestinal tracts [9]. Our study did not include the measurement of serum IgA that may reflect the adherence of *B. hominis* on intestinal epithelial layer. Nevertheless, a past study reported that *B.hominis* infection in mice resulted in predominance of IgA only in intestinal secretions while the serum immunoglobulins were dominated by IgM [47]. A low level of IgA has been reported in humans with *B. hominis* infection [48]. Besides this, insignificant level of serum IgA in patients infected with giardia (a type of intestinal protozoa) has also been observed when comparing with a healthy group [46]. Moving forward, future studies should include IgA in both serum and intestinal secretions in order to study the effect of stress against the pathogenesis of *B. hominis* in terms of damage caused to host's intestinal mucosal layer. This may be further confirmed using histopathological examination of intestinal tissues as well as assessment of protease (enzyme involved in extracellular matrix digestion) activity in intestinal secretions which have been closely associated with invasive pathogens.

In the current study, variation in the levels of total serum immunoglobulins and blood differential count (Figure 3 and 4) in Stressed, Blasto, and Blasto-Stressed groups suggest that stress and *B. hominis* infection independently cause dysregulation of immune system in these rats. Significantly elevated levels of IgG and inflammation associated immunoglobulin, IgE in *B. hominis* infected groups (regardless of stress) compared to the normal group (Figure 3a and 3b) suggest that the parasite attracts these two immunoglobulins to mediate activity of immune cells namely eosinophils in order to combat against the infection. The increased level of eosinophils observed in the Blasto and Blasto-stressed groups supports this speculation (Figure 4d). Furthermore, there are evidences to support the view that IgG [49] and IgE [50] are associated with eosinophil activity to eradicate parasitic infection. In this study, significantly elevated level of serum IgE (Figure 3b) in Blasto-Stressed group compared to Blasto group clearly suggests that stress aggravates the immune dysregulation which may assist in the infectivity of this protozoan as seen in Figure 2. To date there have been no studies done on animal model to evaluate the levels of total serum immunoglobulins in the presence of stress induced *B. hominis* infection. A survey done on 141 leukemic children with high prevalence of *B. hominis* infection has reported that 26% and 41% of them had increased level of serum IgG and IgM respectively [51]. However, the study did not include assessment of leucocytes in these patients. Conversely, in the present study, the IgM level in both Blasto and Blasto-Strssed groups was significantly decreased compared to the non-infected groups (Figure 3c). Similar to our findings, a previous research has shown that serum IgM level in both symptomatic and asymptomatic patients with *B. hominis* were lower than the normal range [48]. These findings suggest that the effects of *B. hominis* infection on total immunoglobulins may vary according to different study population and therefore requires further scrupulous investigations pertaining to immunological pathways. In addition to this, suppressed level of lymphocytes (Figure 4b) and monocytes (Figure 4c) of *B. hominis* infected groups especially in stressed condition (Blasto-Stressed group) leads to the speculation that stress-induced suppression of immune system facilitates the growth of *B. hominis* infection.

Besides this, neutrophils and monocytes are known to be mediators of oxidative stress. However, in the current study there were no changes in the levels of neutrophils especially at week 4 as observed in monocytes. Several *in vitro* studies have shown that the ability of neutrophils (obtained from subjects exposed to brief or maximal exercise) to generate ROS is variable whereby both increase and decrease were evident [52]–[54]. These evidences have indicated that ROS produced during a stress-induced oxidative stress condition may not be totally influenced by the level of neutrophils. In addition, a past study using parasite infected mice model reported that only inflammatory monocytes and not neutrophils were required to combat the infection [55]. In our research, significantly elevated level of monocytes were observed in Blasto-Stressed group in the first week (compared to normal group) which subsequently declined by week 4 (compared to both normal and infected groups). These observations suggest that the monocytes' response in a *B. hominis* infected host could be suppressed when stress is concurrently present.

Investigations on the efficiency of immune system is generally evaluated with the rate of proliferation of lymphocytes (a subset of peripheral blood mononuclear cells) upon the exposure to parasite-specific antigens and non-specific mitogens especially phytohemagglutinin (PHA) [43]. Although immunoglobulin responses are often studied using B cell mitogen namely pokeweed mitogen (PWM), past study has reported that B cells could proliferate in PHA-stimulated lymphocyte cultures [56]. Therefore, in present study PHA was used as positive control to look at the effect of Blasto-Ag towards the growth of PBMCs which consist of both B cells and T cells. Higher level of PBMC proliferation *in vitro* suggests a better establishment of immune responses against the infection [43]. Therefore in this study, we also investigated the effect of solubilized Blasto-Ag on the *in vitro* proliferation level of PBMCs isolated from all four studied groups. Comparison with controls especially in the final week revealed that PBMCs isolated from stressed rats (without infection) had significantly lower level of proliferation and inhibition when stimulated with PHA and Blasto-Ag respectively (Figure 5). This observation supports the general perception that stress is a strong suppressor of immune system thus increasing the susceptibility to infection [43]. Significant inhibition of PBMCs isolated from Blasto group upon exposure to Blasto-Ag (week 1, 2, and 3) clearly suggests that *B. hominis* possesses a very strong ability to suppress immune cell response *in vitro*. This speculation can be supported by our previous *in vitro* study which showed an up-regulation of pro-apoptotic gene in human PBMCs upon exposure to Blasto-Ag [20]. Compared to all other groups, PBMCs isolated from Blasto-Stressed group exhibited highest level of inhibition (week 2, 3, and 4) when stimulated with Blasto-Ag. This result strongly indicates that stress enhances the pathogenecity of *B. hominis* infection by suppressing the activity of host's leucocytes which could be the underlying mechanism for the increasing number of *B. hominis* cysts shown in Figure 2. In addition, the effect of mitogen in these PBMCs was also very minimal when compared to the control group.

As mentioned in the introduction, the process of eradicating an infection involves the host's immune system to produce active oxidants resulting in oxidative damage especially when the amount of antioxidants are lacking. Here we observed that the levels of AOPP and LHP (markers for oxidative protein and lipid damage respectively) in Stressed and Blasto groups were increased compared to the normal group (Figure 6a and 6b). This observation concurs with previous findings which showed that rats subjected to stress [57] and *B. hominis* infection [18] exhibited high levels of protein and lipid oxidations. Interestingly, presence of stress in *B. hominis* infected rats (Blasto-Stressed group) resulted in the elevation of protein and lipid oxidation compared to other groups. In contrast, the levels of both enzymatic and non-enzymatic antioxidants (indicated by FRAP and GPx levels respectively) in the Blasto-Stressed group were significantly lower compared to the normal as well as other groups (Figure 6c and 6d). The decrease of FRAP level in the infected group is in contrast to the observation in our previous study [18]. This disparity could be attributed to the nature of the *B. hominis* isolate used in the previous study (asymptomatic) compared to the symptomatic one used in this study. Such observations suggest that symptomatic *B. hominis* used here could be more pathogenic thus resulting in the suppression of this antioxidant level (vital for combating infections). Reduced levels of both FRAP and GPx in the stressed group of current study suggest that apart from immunosuppression, stress condition mediated the augmentation of *B. hominis* infection by down regulating oxidant-antioxidant balance in the host's system. This theory is further supported by the results obtained from correlation analysis among the oxidative indices as depicted in Table 1. In the normal group, positive correlation was present only between FRAP and GPX implicating that the levels of enzymatic and non-enzymatic antioxidants are always balanced as they are not exposed to any stressors or infections. Whereas in stressed group, correlations of AOPP/LHP, AOPP/FRAP, AOPP/GPx, and FRAP/GPx signify that stress affects the normal oxidant-antioxidant regulatory system in fighting against oxidative damage especially damage towards proteins. Existence of positive correlation of AOPP/GPx in Blasto group shows that enzymatic antioxidants play an important role to combat against the protein oxidation caused by *B. hominis* infection. Conversely, the negative correlation of AOPP/FRAP shows that when stress was present, the oxidative protein damage which resulted due to *B. hominis* infection superseded the level of antioxidant defense mechanism of the hosts.

In conclusion, the present study clearly revealed 3 strong implications: 1) Stress and *B. hominis* infection could independently dysregulate the immune responses as well as the oxidant-antioxidant regulatory systems; 2) Stress enhanced the susceptibility and pathogenicity of *B.hominis* by suppressing or dysregulating responses of PBMC and immunoglobulins; 3) Stress mediated augmentation of *B. hominis* by causing imbalance in the host's oxidant-antioxidant system. The summary of these implications and their relationships are depicted in Figure 7b. The parasite-stress model provided here will serve as a precedence scenario for the better understanding of the role of stress condition in humans against the susceptibility and pathogenicity of *B. hominis* infection.
